# Epidemiological characteristics of pulmonary tuberculosis among health-care workers in Henan, China from 2010 to 2017

**DOI:** 10.1186/s12879-020-05163-8

**Published:** 2020-07-08

**Authors:** Guojie Wang, Jiying Xu, Bin Huang, Sanyou Gao, Yan Zhuang, Kan Wang, Yanqiu Zhang, Jianguo Jiang

**Affiliations:** 1 Medicine Faculty, Henan Medical College, Zhengzhou, Henan China; 2 Anti-TB Institute, Henan Provincial Centre for Disease Control and Prevention, Zhengzhou, Henan China; 3 Pharmacy Faculty, Henan Medical College, Zhengzhou, Henan China

**Keywords:** Pulmonary tuberculosis, Epidemiology, Health-care worker, Occupational exposure

## Abstract

**Background:**

Health-care workers (HCWs) are an epidemiological group with increased exposure to tuberculosis (TB), especially at health-care facilities (HCFs) with poor TB infection control in high-TB-burden settings. China is a high-TB-burden country, and the comprehensive measures for stopping TB transmission at some HCFs were not implemented well owing to limited resources and other factors. The purpose of this study was to review risk of occupational exposure to TB among HCWs and its change trend, and identify epidemiological characteristics of pulmonary tuberculosis (PTB) among HCWs in Henan, central part of China.

**Methods:**

A retrospective cohort study was conducted from 2010 to 2017. All HCWs and teachers in Henan were enrolled to the study as exposed group and non-exposed control group, respectively. Relative risk (RR), attributable risk (AR) and AR percent (AR%) were used to measure the association between the occupational exposure and PTB, and estimated with Poisson regression.

**Results:**

The study results showed a total of 1663 cases of PTB were reported among the HCWs in Henan, accounting for 3.2‰ of all PTB cases reported in the whole population, and annual incidence rate of PTB among HCWs declined by 34% from 2010 to 2017. Over the eight years, the incidence rate of PTB among HCWs was 43.7 cases per 100,000 person-years (PYs), significantly higher than that among teachers (18.8 cases/100,000 PYs), and RR, AR and AR% were estimated to 2.3, 24.9 cases per 100,000 PYs and 57%, respectively. Among HCWs, males were more likely to suffer from PTB than females (adjusted RR: 1.3; 95%CI: 1.2–1.4), and HCWs aged under 25 years had the highest relative risk over all age groups with adjusted RR equaling to 8.3 (95%CI: 6.9-9.9) calculated with those aged 45–54 years as the reference.

**Conclusions:**

Although overall incidence rate of PTB among HCWs showed decreasing temporal trends over the period of 2010–2017, attributable risk of occupational exposure to TB among HCWs did not decrease in Henan, and TB infection at HCFs for males, young or senior HCWs, especially for young HCWs is of much concern.

## Background

Tuberculosis (TB) is still a major public health issue worldwide. Globally, approximately 10.4 million new cases of TB and 1.67 million TB deaths occurred in 2016 [1]. Because of its huge population and high TB incidence rate, China is classified as one of the top 30 high-burden countries of TB, only exceeded by India and Indonesia for the largest number of TB cases [2]. In 2010, the fifth national TB epidemiological survey in China showed that prevalence of PTB was 459 cases per 100,000 population aged more than 15 years old, and farmers accounted for 71.3% of cases [3].

HCWs are an epidemiological group with increased exposure to TB whether in low- or high-TB-burden settings, but the risk increase is much higher at health-care facilities (HCFs) with poor TB infection control in high-TB-burden settings [4–8]. TB infection among HCWs reflects nosocomial TB transmission [9]. TB transmission in health-care settings not only threatens the health of HCWs, and the health of other patients or visitors, especially children or persons living with HIV, diabetes, transplant and other immunosuppressive conditions who access the HCFs, but also increases the chance of two-way spread between health-care settings and communities, bringing the challenge for TB control work in the communities. Moreover, HCWs play a key role in the whole TB control work, and a high burden of occupation-related TB would produce a corresponding negative impact on the health-care workforce, weakening the capability of TB care [10]. It is an indispensable step to block the TB transmission in health-care settings in order to meet the target for “ending the global TB epidemic” [11]. TB transmission at HCFs can be effectively reduced as long as comprehensive measures are taken, including managerial and administrative controls, environmental controls and use of personal protective equipment [12, 13]. However, these measures were not implemented well in developing countries owing to limited resources and other factors [5–7, 14]. China remains similar to other developing countries. One recent nationwide survey covering 12 provinces in China showed that nearly 60% of TB outpatient departments and TB wards had no mechanical ventilation devices installed; one out of three HCWs in TB outpatient didn’t wear N95 respirators, and regular fit test for N95 respirators were only done in 13.7% of health-care institutions; Ultraviolet germicidal devices were not well maintained in spite of being commonly used [15]. Henan was included in this survey and such results reflected the status of Henan to a certain degree.

It is reported that the incidence of PTB among HCWs was lower than that in the general population in China, which is quite different with other countries, so the risk of occupational exposure among HCWs can’t be correctly measured against the general population [16]. The lower TB risk for HCWs probably resulted from a higher socioeconomic status than the general population. In addition, after the National Management Regulation for TB Prevention and Treatment was amended and implemented in 2013, TB infection control work at HCFs improved in Henan, and in recent decades incidence of TB in the general population showed a declining overall trend. With this background, the current status and changing trends for TB burden among HCWs is not well understood. The overall strength of association between occupational exposure and occurrence of TB specifically in Henan remains unclear. The objective of this study was to review risk of occupational exposure to TB among HCWs and its change trend, and identify epidemiological characteristics of PTB among HCWs in Henan of China in order to inform interventions and support future policy-making for reducing TB transmission at HCFs.

## Methods

### Study setting and design

Henan is the third most populous province of China with a population of 95.59 million in 2017, and located in the central part of China. It has 18 administrative divisions including 17 prefecture-level cities and one directly administered county-level city. At the end of 2016, there were 71,273 HCFs including hospitals, clinics, community health service stations and other institutions providing health-care service, and 546,732 HCWs, including all licensed doctors, nurses, pharmacists, and other health-care related technicians working in these institutions. HCWs working in the private clinics were not included.

PTB is one of reportable infectious diseases listed by the Act of People’s Republic of China on the Prevention and Treatment of Infectious Diseases, which classify the reportable diseases into three types, A, B and C. According to the act, PTB is categorized to the type of B, and all PTB cases must be reported within 24 h to local health department and the information related to diagnosis and treatment of these cases are electronically collected by TB Information Management System (TBIMS), which covers the whole country, including Henan. A retrospective cohort study was designed. From January 1, 2010 to December 31, 2017, all HCWs registered in the province were enrolled in the study as the occupationally exposed group, and all teachers registered who had the fulltime teaching position in all education institutions, had the similar status of socioeconomics, in Henan in the same period were selected as the non-exposed control group. The risk of occupational exposure was estimated as the incidence rate of PTB among HCWs against incidence rate of PTB among teachers.

### Data sources

All data of PTB cases were extracted from TBMIS in Henan Provincial Center for Disease Control and Prevention. All PTB cases included in this study met the Standard for Pulmonary TB Diagnosis (WS288–2008) issued by the former Ministry of Health, the People’s Republic of China in 2008. Demographic data of HCWs were obtained from the Statistical Information Centre, Henan Provincial Health Commission. The population data (2010–2017) for the teachers and the whole province were from Henan Statistical Yearbook (2017) and website of National Bureau of Statistics of China (http://data.stats.gov.cn/).

### Statistical analysis

The distribution of demographic characteristics of PTB cases among HCWs was described. Incidence rates of PTB were used to measure TB burdens among HCWs, teachers and in the whole population, and the comparisons between them were conducted. Temporal trends of annual incidence rates of PTB among HCWs, teachers and in the whole population were analyzed with simple linear regression, respectively, and the correlations between the annual incidence rates among these three groups were measured with Pearson correlation coefficients. Relative risk (RR) and its 95% confidence interval (95%CI) were used for measuring the association between occupational exposure and PTB regarding incidence rate of PTB among HCWs against the incidence rate of PTB among teachers. Attributable risk (AR) is used to quantify the risk that can be attributed to occupational exposure in HCWs and it was measured with the difference of incidence rates of PTB between HCWs and teachers. AR and AR percent (AR%) were calculated as AR = I_H_-I_t_, AR% = 100*(I_H_-I_t_)/I_H_, where I_H_ = incidence rate among HCWs, and I_t_ = incidence rate among teachers. Moreover, RR and its 95%CI used to measure the association between demographic factors and PTB among HCWs were estimated with Poisson regression. Incidence rate was calculated as: 100, 000*number of cases/number of years observed. An approximate method is used for calculating the number of person years (PYs): number of PYs = sum of (annual average number of persons observed). The data process and statistical analysis were conducted by SPSS (version 24). Purely spatial scan statistical analysis for clusters with high rates was conducted through the geographical regions of the 18 administrative divisions mentioned above with the software of SaTScan (version9.6). During the analysis, the discrete Poisson model was applied to identify the geographic areas of potential clusters having the number of cases reported, which exceeded the number of cases expected with statistically significance (*p* < 0.05).

### Ethics statement

This study was approved by the Institutional Review Board of Henan Provincial Centre for Disease Control and Prevention. All data were de-identified and confidentiality of data was maintained.

## Results

### Cases, incidence rates and RRs of PTB among HCWs by year

**Fig. 1:**
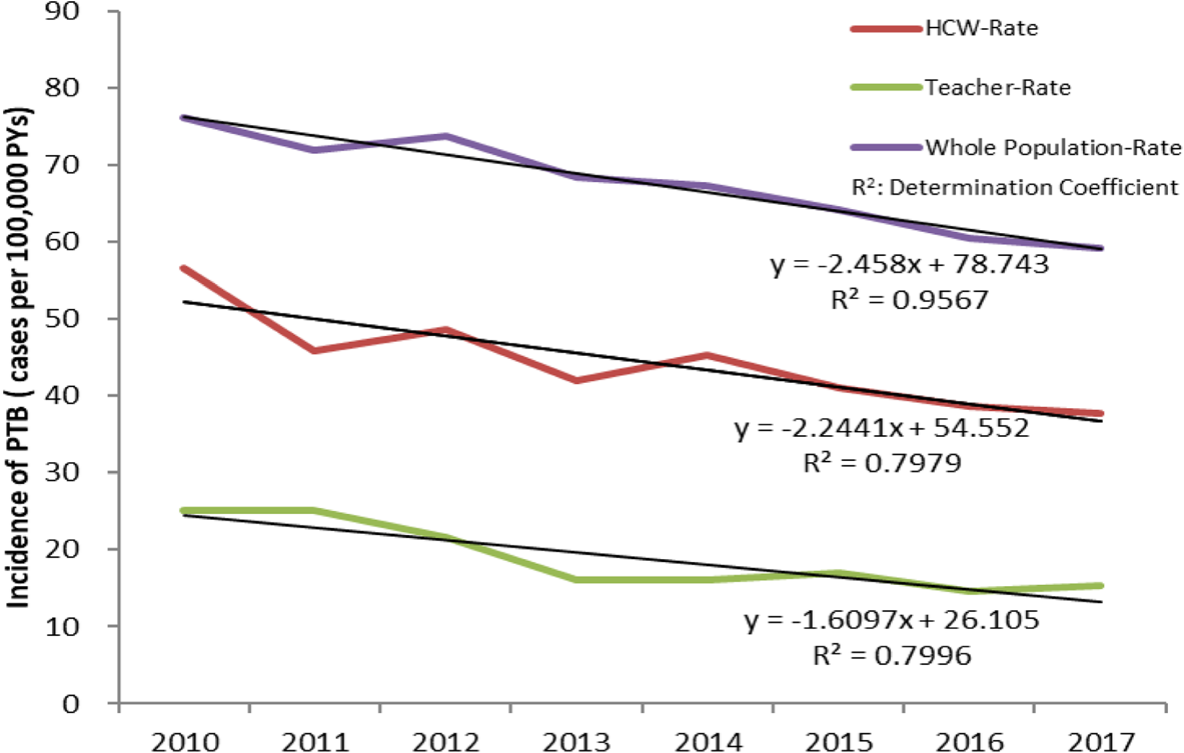
Incidence rates of PTB among HCWs, Teachers and in the whole population by year, Henan of China, 2010–2017

In total, 1663 cases of PTB diseases were diagnosed and reported among HCWs in Henan over the periods from 2010 to 2017, accounting for 3.2‰ (1663/511699) of all PTB cases reported in the whole population. The annual incidence rates of PTB among HCWs showed a decreasing temporal trend over the period of 2010–2017, similar to the temporal trend of incidence rates among teachers and in the whole population, but downward speeds were different. Annual incidence rate among HCWs declined by 34% from 56.6 cases per 100,000 PYs in 2010 to 37.3 cases per 100,000 PYs in 2017. In comparison, annual incidence rates of PTB among teachers and in the whole population decreased by 39 and 22%, respectively. The simple linear regression models used to estimate the relationship between the three annual incidence rates and time period all demonstrated that they fitted well based on the coefficients of determination (all R^*2*^ > 0.7). The annual incidence rates of PTB among HCWs were correlated with those among teachers and among the whole population with Pearson’s correlation coefficient (r) equaling to 0.8 and 0.9, respectively (both *p* < 0.05). (Table 1, Fig. 1).

**Table 1:** Rate and cases of PTB among the whole population, HCWs and Teachers, and proportion of PTB reported among HCWs against the total PTB cases reported among the whole population, by year in Henan of China, 2010–2017

Year	Whole Population			HCW			Teacher			HCW-Case Proportion (‰)
	n (10,000)	Cases	Rate (/100,000)	n (10,000)	Cases	Rate (/100,000)	n (10,000)	Cases	Rate (/100,000)	
2010	9405	71,681	76.2	37.28	211	56.6	101.22	254	25.1	2.9
2011	9388	67,568	72.0	39.63	182	45.9	102.56	258	25.2	2.7
2012	9406	69,413	73.8	42.85	208	48.5	103.30	223	21.6	3.0
2013	9413	64,326	68.3	46.85	197	42.0	102.93	165	16.0	3.1
2014	9436	63,487	67.3	49.48	224	45.3	103.87	169	16.3	3.5
2015	9480	60,825	64.2	51.99	213	41.0	105.40	182	17.3	3.5
2016	9532	57,657	60.5	54.70	211	38.6	108.97	158	14.5	3.7
2017	9559	56,742	59.4	58.10	217	37.3	111.27	170	15.3	3.8
Total	75,619	511,699	67.7	380.88	1663	43.7	841.31	1579	18.8	3.2

Over the eight years from 2010 to 2017, the incidence rate of PTB among HCWs was 43.7 cases per 100,000 PYs, significantly higher than the rate among teachers (18.8 cases /100,000 PYs), but significantly lower than the rate in the whole population (67.6 cases /100,000 PYs). Health-care occupational exposure was highly associated with an increased risk of PTB with RR equaling to 2.3 (95%CI: 2.2–2.5). AR and AR% were estimated to be 24.9 cases per 100,000 PYs and 57%, respectively. Compared with 2010–2013, RR in 2014–2017 showed a slight increase (2010–2013: 2.2; 2014–2017: 2.6), and AR% had a similar trend (2010–2013: 54.1%; 2014–2017: 61.2%) although incidence rate of PTB among HCWs showed a decreasing trend. (Table 2).

**Table 2:** Incidence rates of PTB among HCWs and teachers, RR, AR and AR%, Henan of China, 2010–2013 and 2014–2017

Year	HCW			Teacher			RR (95%CI)	AR (/100,000 PYs)	AR%
	PYs (10,000)	Cases	Rate (95%CI) (/100, 000 PYs)	PYs (10,000)	Cases	Rate (95%CI) (/100,000 PYs)			
2010–2013	166.61	798	47.9 (44.7–51.3)	410.01	900	22.0 (20.6–23.4)	2.2 (2.0–2.4)	25.9 (24.2–27.9)	54.1
2014–2017	214.27	865	40.5 (37.9–43.3)	429.51	679	15.7 (14.6–17.0)	2.6 (2.3–2.8)	24.8 (23.2–26.3)	61.2
2010–2017	380.88	1663	43.7 (41.7–45.9)	839.52	1579	18.8 (17.9–19.7)	2.3 (2.2–2.5)	24.9 (23.8–26.2)	57.0

### Sex and age distribution

Of the total 1663 PTB cases among HCWs, 64.5% of them were female, and the group aged less than 35 years accounted for 64.7%. With age increasing, the proportion of PTB cases by age group showed a decreasing trend other than the group aged less than 25 years. Incidence rate of PTB in male HCWs were significantly higher than in females (49.4 vs 41.1 cases per 100,000 PYs, *p* < 0.05). Across all age groups, less than 25-year-old HCWs had the highest incidence rate (174.4 cases per 100,000 PYs), and the group aged 45–54 years had the lowest incidence rate (22.7 cases per 100,000 PYs). Males and persons aged under 35 years old or above 54 years old among HCWs had higher risk associated with PTB with statistical significance (*p* < 0.05). Particularly, persons aged less than 25 years old among HCWs had the highest risk over all age groups. RR and 95% CI for persons aged under 25 years were 7.7 (6.4–9.2) regarding the group aged 45–54 years as the reference. After being adjusted, sex and age were still associated with the occurrence of PTB with statistical significance (*p* < 0.05). Using 2010–2013 as reference, adjusted RR for the time period of 2014–2017 was 0.86 (95%CI: 0.78–0.94), meaning that even after removing the effect of age and sex there was still a decrease of 14% in the risk of TB in HCW in the period 2014–2017. No interaction was found between sex and age. (Table 3).

**Table 3:** Incidence rate of PTB among HCWs and RR by sex, age and year, Henan of China, 2010–2017

Demographics	PYs	Cases	%	Rate (95%CI) (/100,000 PYs)	RR (95%CI)	Adjusted RR (95%CI)
Sex						
Male	1,195,674	591	35.5	49.4 (44.7–54.6)	1.2 (1.1–1.3)	1.3 (1.2–1.4)
Female	2,607,626	1072	64.5	41.1 (38.7–43.6)	Reference	Reference
Age (years)						
< 25	197,212	344	20.7	174.4 (145.2–209.6)	7.7 (6.4–9.2)	8.3 (6.9–9.9)
25–34	1,509,921	732	44.0	48.5 (41.0–57.3)	2.1 (1.8–2.5)	2.2 (1.9–2.6)
35–44	1,080,137	269	16.2	24.9 (20.6–30.2)	1.1 (0.9–1.3)	1.1 (0.9–1.3)
45–54	749,250	170	10.2	22.7 (19.5–26.6)	Reference	Reference
> =55	266,780	148	8.9	55.5 (44.5–69.2)	2.4 (2.0–3.0)	2.3 (1.9–2.9)
Year						
2010–2013	1,666,050	798	48.0	47.9 (43.5–52.8)	Reference	Reference
2014–2017	2,137,190	865	52.0	40.5 (37.9–43.3)	0.85 (0.77–0.91)	0.86 (0.78–0.94)

### Distribution of PTB among HCWs by administrative divisions

**Fig. 2:**
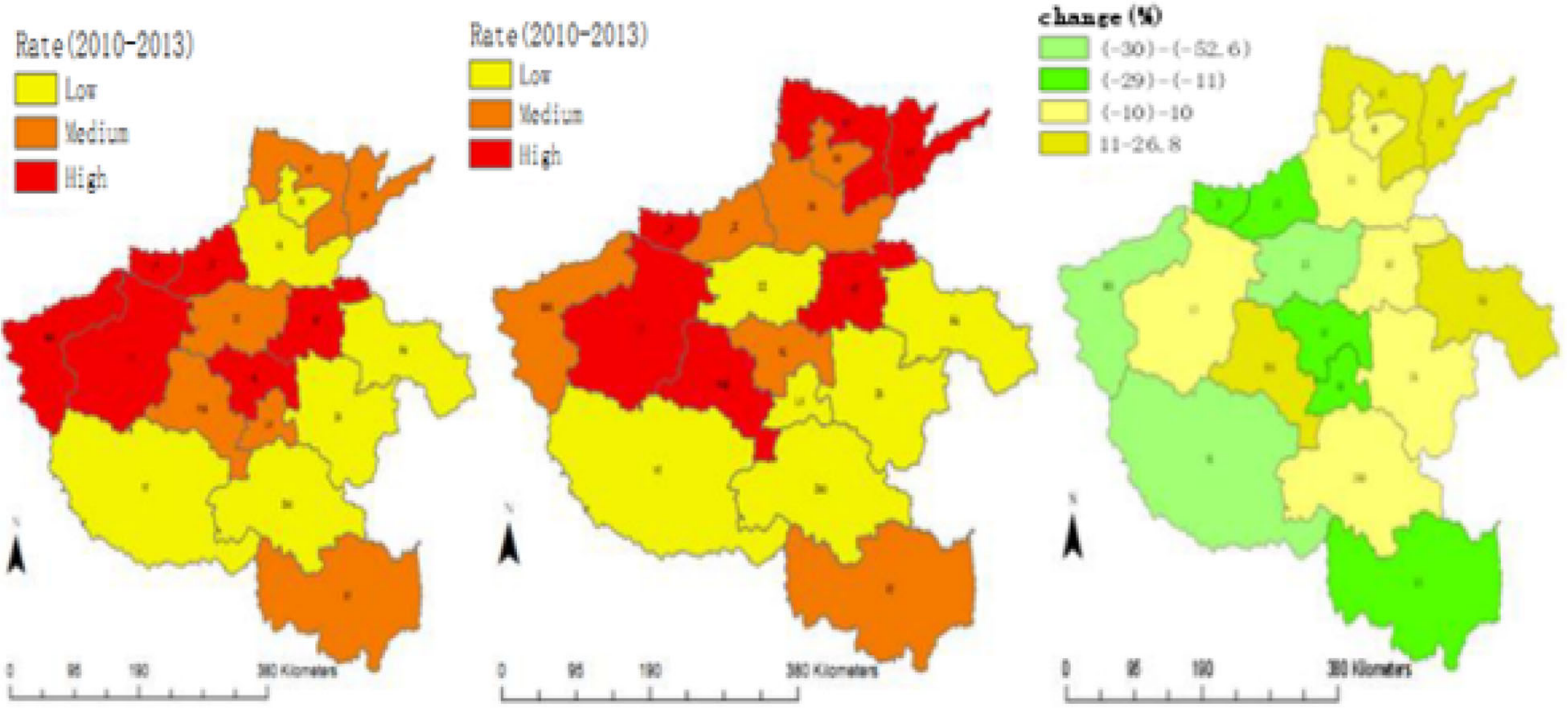
Geographic distribution of incidence rate and change of PTB among HCWs, Henan of China, 2010–2013 and 2014–2017

In 2014–2017, incidence rate of PTB among HCWs in the 18 administrative divisions ranged from 21.2 to 88.9 cases per 100,000 PYs. Comparing with the period of 2010–2013, the incidence rates in 12 administrative divisions decreased by 3.7–52.6%., and in the other six administrative divisions increased by 3.7–26.8%. Based on the tercile of the incidence rate in each time period, the cities were categorized into three levels with low, medium and high incidence rates, respectively. In 2010–2013, the cities with high incidence rate were aggregately located in two areas, one in the northwest of the province and another in the core area, and in 2014–2017 the pattern was slightly changed. The original two areas with high incidence rates were shrunk and one new area with high incidence rate appeared in the north. (Fig. 2).

### Spatial clustering analysis

**Fig. 3:**
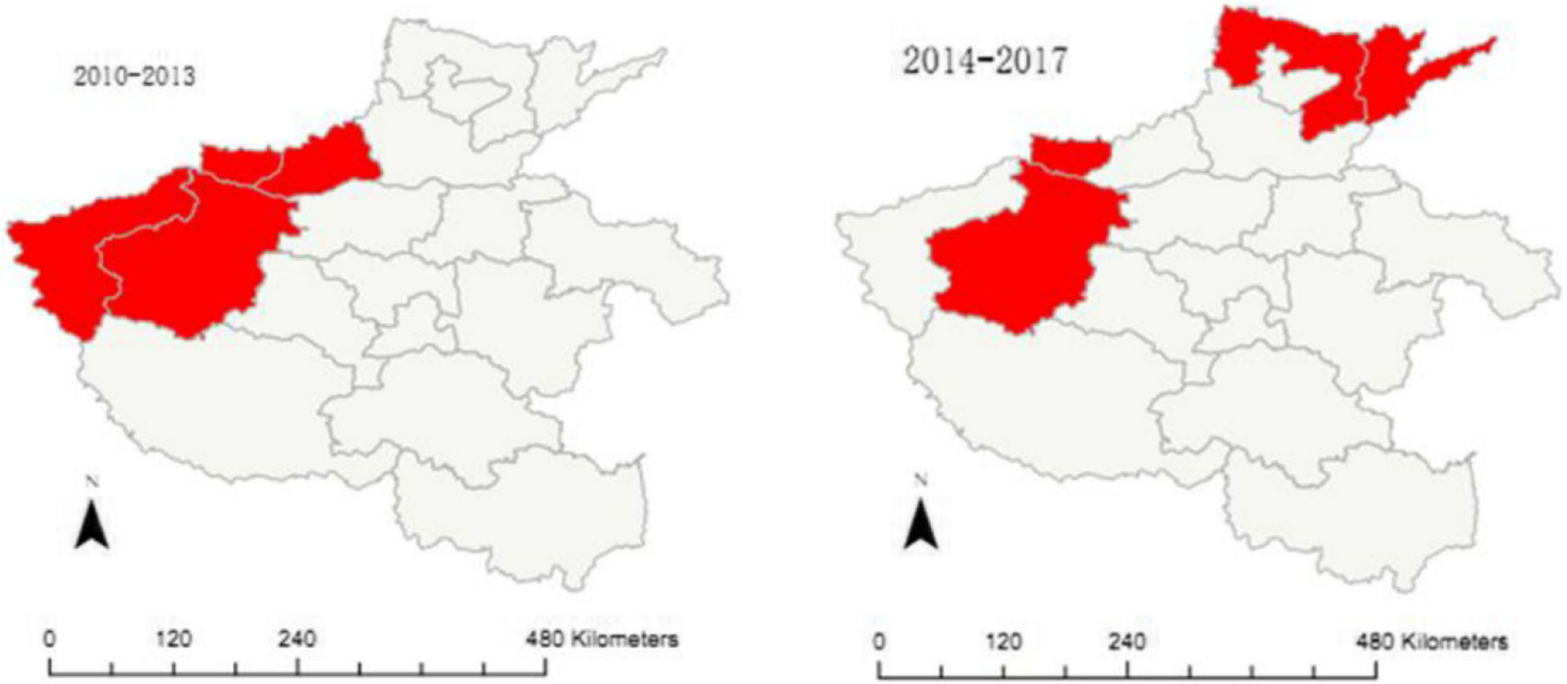
Geographic distribution of clusters detected with spatial analysis scanning for high-rate of PTB among HCWs, Henan of China, 2010–2013 and 2014–2017

Purely spatial analysis scanning for high-rate clusters using the discrete Poisson model with software of SaTScan was conducted for the time periods of 2010–2013 and 2014–2017, respectively. In the period of 2010–2013, one cluster was detected, including four administrative divisions in the northwest of the province. This cluster still existed in the period of 2014–2017, but it was shrunk to an area including two administrative divisions; another cluster including two administrative divisions in the north was detected in the period of 2014–2017. (Table 4, Fig. 3).

**Table 4:** Cluster analysis results for the two time periods, 2010–2013 and 2014–2017

Cluster	administrative divisions included	Observed Cases(n)	Expected cases(n)	Rate (cases/10^5^ PYs)	Radius (43.86 km)	RR	LLR	*P*-value
1 (2010–2013)	SMX,LY,JY,JZ	188	123	70.9	191.45	1.7	17.93	< 0.001
2 (2014–2017)	JY,LY	123	75	65.0	43.86	1.8	14.54	< 0.001
3 (2014–2017)	PY,AY	106	77	54.4	72.05	1.4	5.48	0.03

## Discussion

The results in this present study demonstrated that having a high-level of socioeconomic status, HCWs had a lower incidence of PTB in Henan than the general population despite the fact that they were faced with additional risk from occupational exposure, but compared with teachers who had similar socioeconomic status, the incidence of PTB among HCW increased 2.3 times; among HCWs, sex and age were independently associated with the incidence of PTB, and the incidence rate of PTB among HCWs showed a decreasing trend before and after adjusted with sex and age in Henan. In this present study, HCWs had a lower incidence than general population consistent with previous report [17]. In 2016, WHO reported that 16 countries had ratios more than 2 for TB incidence rate among HCWs compared to the rate in the general population, three countries had the ratio between 1 and 2, and China had the ratio below 1 [17]. Most previous studies indicated that HCWs had higher incidence rate than the general population although there were some exceptions as well [16, 18, 19]. The value of AR in this present study was estimated to 24.9 cases per 100,000 PYs in 2010–2017, suggesting that currently about 140 cases occurred in Henan each year due to occupational exposure only. Based on the value of AR% in 2010–2017 in this present study, 57% of HCW TB cases resulted from occupational exposure in Henan. By comparison, in a meta-analysis it was estimated that HCW occupational exposure AR% in the countries with low (< 50), intermediate (50–100) and high incidence of TB (> 100 cases per 100,000 persons) were 49, 27 and 81%, respectively [16]. TB infection among HCWs can be from both sources of HCFs (occupational exposure) and communities (non-occupational exposure). It is critical to choose an appropriate non-occupational exposure group when the risk attributed to occupational exposure is estimated. Most studies chose the general population. In Henan, farmers accounting for the largest proportion of whole population, have a quite different socioeconomic status with HCWs. If the general population was chosen as non-occupational exposure group, the risk of TB attributed to the health-care occupational exposure would be underestimated since it is well-known that socioeconomic status is strongly associated with TB. It is more reasonable to measure occupational exposure at HCFs with indicators of AR and AR% using teachers as non-occupational exposure group due to the similar socioeconomic status in Henan. Additionally, one study based on DNA fingerprints of the bacteria profile reported that 24% of HCW TB cases were infected during work in a setting with a low incidence of TB, which was lower than this present study [20].

The incidence rate of PTB among HCWs showed a decreasing trend in Henan in this present study, and it still remained the same trend after adjusted with sex and age. There is no doubt that efforts to stop TB nosocomial infection reduced the risk of occupational exposure, leading to decreasing trend of PTB among HCWs, but probably it also played an important role in Henan that the incidence of PTB in the general population decreased. With the decreasing incidence of PTB in the general population, the number of patients with PTB seeking service in health-care settings would decline, which would result in a less HCW exposure. Over the eight-year period of the study, the incidence rates of PTB between HCWs and general population (the whole population) in Henan were strongly correlated (Pearson Correlation Coefficient = 0.9). In addition, comparing with the period of 2010–2013, HCW occupational RR and AR, estimated with the teachers as unexposed group, had no significant change in the period of 2014–2017 (95%CI for RR: 2.0–2.4 vs 2.3–2.8; 95%CI for AR: 24.2–27.9 vs 23.2–26.3). They support that the decreasing burden of PTB in the general population in the communities probably contributed to the decreasing trend of PTB incidence among HCWs in Henan.

Of all cases of PTB among HCWs, females predominated. However, males had a higher incidence rate of PTB than females, suggesting that males were more likely to suffer from PTB among HCWs than females. It was consistent with the general population and other previous study on HCWs [21, 22]. The national TB survey in China also demonstrated that the prevalence of TB in males of all age groups were higher than that in females (*p* < 0.05) [3]. An interesting finding from this study is the association between age and PTB among HCWs. The young HCWs aged less than 25 years had the highest risk across all age groups. For a further analysis, we compared the incidence rate of PTB among HCWs aged less than 25 years with that in the whole population aged 15–24 years over the same period, 2010–2017, which were 174.4 cases per 100,000 PYs and 81.2 cases per 100, 000 PYs, respectively. The former was 2.1 times as high as the latter. Young HCWs are new employees in health-care settings, having a short-term occupation exposure and lacking of knowledge and awareness of personal protection against TB nosocomial infection. Most of them are susceptible to TB infections. If the risk of TB transmission at HCFs is high, the infection attributed to occupational exposure probably occurred within the first several years after they start to work at HCFs. About 10% of persons infected with TB progress to active disease during their life time, but the progression risk is highest in the first 1–2 years after infection [23]. As a high-risk group for TB, young HCWs are an important part of nosocomial infection control. Training for young HCWs will raise their awareness of personal protection against TB transmission at HCFs.

As for the geographic distribution, PTB incidence among HCWs in Henan varied with the administrative divisions in both periods, and the overall distribution patterns were similar between the two periods. Despite that overall incidence of PTB among HCWs showed declining, not all administrative divisions saw the declining. On the contrary, incidences of PTB among HCWs in several administrative divisions increased. These findings remind that maybe the risk of PTB attributed to the occupational exposure was varied at the administration division level with different settings of TB incidence in the communities and different measures taken for TB infection control at HCFs. Most administrative divisions with higher-level incidence rates among HCWs were located in the north. The spatial clusters detected with high incidence were consistent with the analysis of geographic distribution. The administrative divisions covered by the clusters all had the high-level incidence rates of HCWs PTB. The clusters identified were all located in the northwest or north of the province. None of these clusters identified were located in the area with a higher incidence of TB among general population, which was usually referred to as the south of the province [24]. Comparing with spatial patterns of socio-economic developing status reported in Henan [25], none of these clusters occurred in the administration divisions with the low-level status of socio-economic development either. It sounds that TB incidence among the general population and socio-economic developing status were not spatially correlated with the clusters. It needs to conduct whether areas covered by the clusters identified all had poor TB control measures implemented and an increased risk attributed to the occupational exposure.

This present study has some limitations. Extrapulmonary TB was not included since it is not reportable bylaw and the data was not collected bylaw. This would underestimate the TB burden and the risk attributed to the occupational exposure among HCWs. The data on PTB cases and the population of HCWs were sourced from different information systems, so perhaps there were slight gaps for counting. HCWs working in the private clinics and the persons who used to work as HCW or teacher were not included. These limitations can lead to a bias of the estimates. Since this is a retrospective study based on TBIMS, the related information was not enough to provide more details analysis on some potential factors impacting the TB risk attributed to the occupational exposure, e.g. position of HCWs, implementation status of the measures taken to stop TB infection at HCFs. The spatial clustering analysis only exhibited the distribution of the clusters with significantly higher incidences of PTB among HCWs across the administration divisions, and no further analysis was conducted to explore whether they were spatially correlated to the distribution of the increased risk factors like shortage of resources allocated on nosocomial TB infection control, poor implementation status of measures taken for stopping infection at HCFs owing to the failure to collect the data. The incidences estimates used in this study were based on the notified cases collected by TBIMS. This probably caused an underestimation as well, but the underestimation should be very small because the detection rate of TB among the general population has reached a high level in Henan since the DOTS strategy was implemented in the late 1990s. Our results showed the incidence rate of PTB in the general population in Henan was 59.2–76.2 cases per 100,000 persons, consistent with the incidence rate in 2016 which was estimated to be 64 (55–74) cases /100,000 in China by WHO [2]. Owing to good education, high-income occupations and knowledge of TB, both HCWs and teachers have good access to health services. After developing the symptoms of PTB, they can be diagnosed and reported quickly. In addition, in order to avoid missing reporting and management, it has been enforced that only the health-care institutions assigned by the health department can provide PTB treatment and management in Henan since late 1990s. Therefore, the underreporting should be very low.

## Conclusions

The incidence rate of PTB among HCWs in Henan demonstrated a falling trend, and remained much higher than that among teachers, who have similar socioeconomic status as HCWs.; more than a half of PTB cases among HCWs were attributed to occupational exposure at HCFs, and the attributable risk of occupational exposure to TB among HCWs did not decline in Henan over the study period; TB infection at HCFs for males, young or senior HCWs, especially for young HCWs is of much concern.

## Data Availability

All data supporting the findings can be found in this article.

## Abbreviations

HCWs: Health-care workers
TB: Tuberculosis
HCFs: Health-care facilities
PTB: Pulmonary tuberculosis
PYs: Person years
RR: Relative risk
AR: Attributable risk
CI: Confidence interval
TBIMS: TB Information Management System
